# Total Worker Health Leadership and Business Strategies Are Related to Safety and Health Climates in Small Business

**DOI:** 10.3390/ijerph17062142

**Published:** 2020-03-24

**Authors:** Natalie V. Schwatka, Miranda Dally, Liliana Tenney, Erin Shore, Carol E. Brown, Lee S. Newman

**Affiliations:** 1 Center for Health, Work & Environment and Department of Environmental and Occupational Health, Colorado School of Public Health, University of Colorado, Anschutz Medical Campus, 13001 E. 17th Pl., 3rd Floor, Mail Stop B119 HSC, Aurora, CO 80045, USA; miranda.dally@cuanschutz.edu (M.D.); liliana.tenney@cuanschutz.edu (L.T.); erin.shore@cuanschutz.edu (E.S.); carol.brown@cuanschutz.edu (C.E.B.); lee.newman@cuanschutz.edu (L.S.N.); 2 Department of Epidemiology, Colorado School of Public Health, University of Colorado, Anschutz Medical Campus, 13001 E. 17th Pl., 3rd Floor, Mail Stop B119 HSC, Aurora, CO 80045, USA; 3 Department of Medicine, School of Medicine, University of Colorado, Anschutz Medical Campus, 13001 E. 17th Pl., 3rd Floor, Mail Stop B119 HSC, Aurora, CO 80045, USA

**Keywords:** Total Worker Health, safety climate, health climate, small business leadership, safety leadership, health leadership, occupational safety and health, worksite wellness

## Abstract

The purpose of this study was to investigate the relationship between Total Worker Health^®^ (TWH) business strategies and employee perceptions of leadership commitment and safety and health climates. Using data from 53 small enterprises and 1271 of their workers collected as part of the Small + Safe + Well (SSWell) Study, we confirm the primacy of the relationship between leadership commitment to safety and workplace safety climate. After accounting for leadership commitment to safety, business-reported policies and practices that promote the health, safety, and well-being of workers (i.e., TWH strategies) were no longer related to safety climate. In contrast, the relationship between TWH strategies and health climate were significantly associated with the level of small business leadership commitment to worksite wellness. Relatedly, our results demonstrate that leadership is a common correlate to both safety climate and health climate. Future research should investigate integrated TWH leadership development strategies as a means of simultaneously improving safety and health climates.

## 1. Introduction

The United States National Institute for Occupational Safety and Health (NIOSH) defines Total Worker Health^®^ (TWH) as policies, programs, and practices that integrate protection from work-related safety and health hazards with the promotion of injury and illness prevention efforts to advance worker well-being [1]. This framework prioritizes changes to the work environment such that workers are able to be physically and psychosocially healthy. We chose to focus on three key indicators of the work environment in the present study. We first evaluate business TWH strategies as they reflect business-reported policies and practices that promote the health, safety, and well-being of workers. We also investigate safety and health climates, which are defined generally as employee perceptions that their organization cares about their safety and their health and well-being, respectively. These climates represent the gut check between what business TWH strategies are in place and how supported they are by leadership and the organization in values and day-to-day activities. Finally, we evaluate leadership commitment to safety and leadership commitment to health to understand how they are associated with the implementation of TWH strategies in day-to-day practice. The purpose of this paper is to evaluate the relationship between TWH business strategies and safety and health climates and whether this relationship is moderated by leadership commitment and to do so in an understudied population: small businesses.

Small businesses represent the majority of firms in the United States. In 2016, 47% of people in the United States were employed by businesses with fewer than 500 employees with over two-thirds (70%) of them working for businesses with fewer than 100 employees [2]. This population has a significant number of work-related injuries, illnesses, and fatalities as well as poor health and wellness [3]. Small businesses often struggle to create and implement programs to protect and promote worker health due to lack of knowledge, resources, and competing priorities [4]. For example, in our research with 382 small businesses, we found that smaller businesses consistently scored lower on an organizational assessment of TWH policies and programs than larger businesses [5]. There is a paucity of literature regarding the factors that contribute to small business safety and health environments, including safety and health climates [6] and how they relate to TWH strategies and leadership practices.

### 1.1. Safety and Health Climates

Organizational climate perceptions stem from shared employee perceptions of the work environment and observations about what kinds of behaviors get rewarded and supported on the job [7,8]. Organizations have many climates; each focused on a specific facet of the workplace environment. After decades of investigating these climates in silos, Schneider [9] argues that it is time to evaluate them in tandem. This is in line with the TWH framework, which advocates for the integration of business functions to address more complex workforce safety, health, and well-being issues [10]. To begin to address this, we have argued for the importance of evaluating safety climate and health climate in the context of TWH [11].

With regard to safety climate, almost four decades ago, Zohar [12] published the first safety climate study and demonstrated that employees agreed on the relative importance of safe work behavior and that this was related to worksite safety practices. Since this seminal study, researchers have evaluated the relationship between safety climate and safety outcomes. Meta-analyses demonstrate that safety climate is significantly related to safety motivation, knowledge, behavior, and accidents [13,14,15,16]. Thus, there is evidence that safety climate, defined as employee perceptions of their organization’s commitment to workplace safety programs and their beliefs about how much their organization values having a safe workplace, is correlated with numerous worksite safety indicators.

Organizations also have a climate for health, which is defined as employee perceptions of their organization’s commitment to the physical and psychological health of employees. The distinction between safety climate and health climate is important, because they likely represent conceptually distinct constructs [17]. A business may have supportive safety policies but may lack supportive health-promoting policies, or vice versa [18]. Indeed, Basen-Engquist et al., [19] found that a health promotion intervention had a positive impact on health climate but not on safety climate. Support for the relationship between health climate and employee health outcomes, such as physical health symptoms and health behaviors, has been documented in the literature [18,19,20].

### 1.2. Predictors of Safety and Health Climates

Researchers rarely study the factors that may lead to positive safety climate and health climate perceptions [21,22]. Climate stems from employees observing their work environment and discerning the relative priorities over other competing demands. As such, employees’ perceptions of safety climate and health climate reflect employees trying to make sense of consistencies or inconsistencies in what TWH policies and programs their organization adopts and how they are applied on a daily basis. In other words, climate not only reflects existing TWH policies and programs but also the perceived value and importance of safety and health within the organization.

#### 1.2.1. TWH Strategies

**Figure 1:**
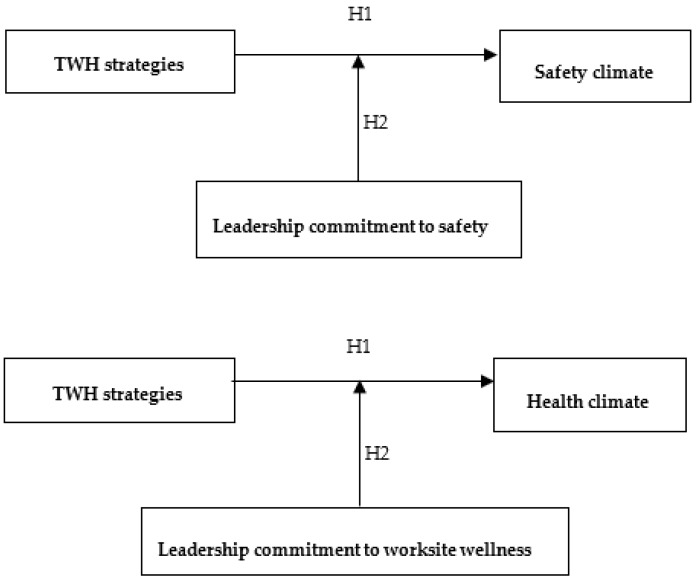
Hypothesized relationships between Total Worker Health^®^ (TWH) strategies (benchmarks).

Business policies and practices that support employee safety, health, and well-being serve as one cue to employees that they are supported when they act in ways that protect and promote their health [23]. In a review of the safety climate intervention literature, Lee et al. [24] found that many of the successful interventions that demonstrated improvements in safety climate included changes to the organizational and managerial structure, job design, personnel and training, and the psychosocial and physical work environment. Thus, we hypothesize that TWH strategies, which are defined as the business-reported policies and practices that are designed to promote the health, safety and well-being of workers, are significantly related to safety climate and health climate perceptions (see Figure 1). There are several systematic TWH strategies that employers can use to protect and promote employee health, all of which share common elements or benchmarks. For example, Tenney et al. developed the Health Links™ Healthy Workplace Assessment, which is a six-benchmark organizational-level assessment instrument that has been successfully utilized by small businesses [5] and that we used in the current study.

**Hypothesis** **1.** *Small businesses that have more TWH strategies have better safety and health climates than small businesses that have fewer TWH strategies.*

#### 1.2.2. Moderating Effects of Leadership Commitment

The literature describing predictors of climate focuses almost exclusively on leadership as a major driver of organizational climate. While TWH strategies may play a role in the development of safety climate and health climate perceptions, it is likely that it is the consistency with which they are implemented by leadership that results in the development of climate perceptions [21]. Leaders may or may not implement TWH strategies by attending to, rewarding, monitoring, and talking about TWH strategies with their employees [9]. As employees observe these actions, they evaluate the priority of TWH strategies against other competing organizational priorities and make judgments about how they should act at work.

In the occupational safety literature, meta-analyses find that leadership is significantly related to safety climate perceptions [13,14,25]. Researchers demonstrate that active forms of leadership exhibit a positive relationship to safety climate, while passive forms of leadership exhibit the opposite effect [26]. They have also shown that employees who report that their leaders inconsistently support safety report poorer safety climate perceptions [27]. The safety intervention research literature demonstrates that improving safety leadership results in improvements in safety climate perceptions [28,29,30]. To our knowledge, there is no literature evaluating the relationship between health-promoting leadership and health climate; however, we would expect to observe similar relationships.

Thus, we hypothesize that leadership commitment moderates the relationship between TWH strategies and safety and health climates (see Figure 1). Employees who work for businesses with more TWH strategies and who report excellent leadership commitment to safety and worksite wellness are likely to report a more positive safety climate and health climate. On the other hand, employees who work for businesses with more TWH strategies but who report poorer leadership commitment to safety and worksite wellness are likely to report a less positive safety climate and health climate.

**Hypothesis** **2.** *The relationship between TWH strategies and safety and health climates in small businesses is moderated by leadership commitment to safety and leadership commitment to worksite wellness.*

### 1.3. Study Purpose

The purpose of this study was to evaluate whether leadership commitment to safety and worksite wellness moderated the relationship between TWH strategies and safety and health climates in small businesses. It is important to evaluate these relationships amongst small businesses. They typically have few resources for TWH, and their employees bear a disproportionate burden of injuries, illnesses, and fatalities [31]. There is also a paucity of research on TWH strategies, leadership, and climate in small organizations [6,11]. Our research amongst small businesses suggests that there is a significant amount of variability in the implementation of TWH strategies amongst small businesses [5]. Our next step is to evaluate whether these strategies are related to leadership and climate in a cohort of small businesses.

## 2. Materials and Methods

### 2.1. Participants

#### 2.1.1. Businesses

The organizations included in this study are from the Small + Safe + Well (SSWell) study. The SSWell study is a TWH intervention study that aims to assess improvements in TWH workplace practices, health and safety climate, and ultimately health, safety, and well-being in small businesses across the state of Colorado [11]. The intervention includes participation in Health Links™ and a TWH leadership program, both of which are described in the research of Schwatka et al. [11].

**Figure 2:**
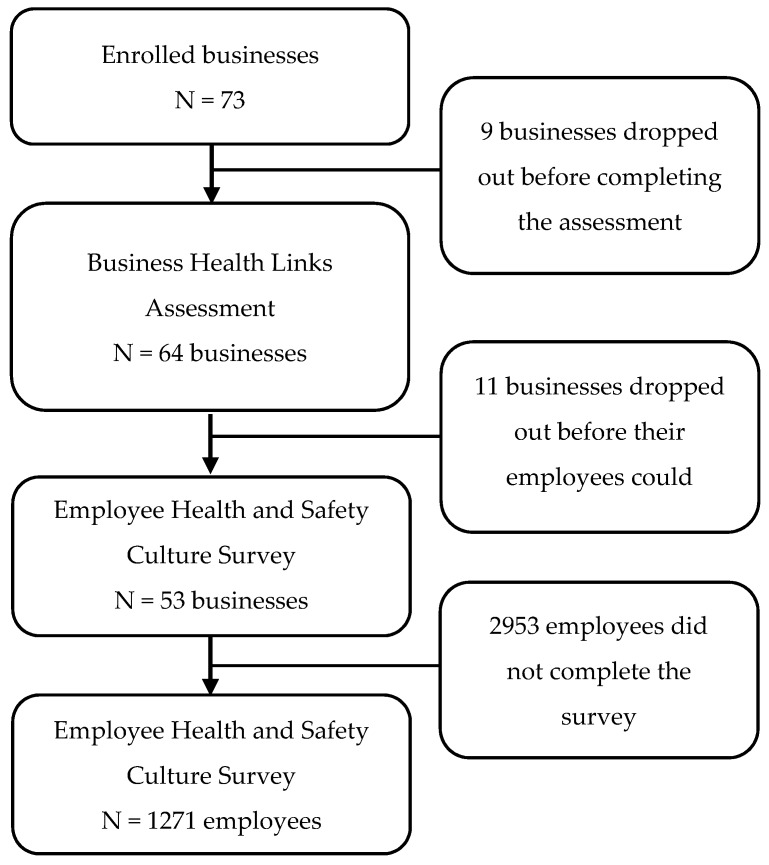
Study enrollment and flow.

We recruited businesses through outreach efforts, including email marketing, events, trainings, and channel partners. These groups included chambers of commerce, workers’ compensation insurers, local public health agencies, health and wellness coalitions, and trade associations. Additionally, organizations that enrolled in Health Links within the month prior to our SSWell recruitment period were invited to participate. Businesses received free services including Health Links™ and TWH leadership program participation as an incentive to participate. Businesses were eligible to participate if they (1) had fewer than 500 employees, (2) were an established company, non-profit, government agency, or other type of organization, and (3) were operating in Colorado. Seventy-three businesses in the present study enrolled between April 2017 and December 2018, and 53 businesses completed all the required study assessments (see Figure 2).

#### 2.1.2. Employees

Once a business agreed to participate and had completed the Health Links Healthy Workplace Assessment™, their employees were invited to complete an online employee health and safety culture survey via email. Employees reviewed an informed consent page prior to beginning the anonymous online survey. Completion of the survey indicated their consent to participate in the study. Ultimately, 1271 employees completed the employee health and safety culture survey (see Figure 2). This study was approved by the Colorado Multiple Institutional Review Board (COMIRB).

### 2.2. Data Collection and Measures

For the purpose of this study, we utilized cross-sectional data collected at the business and employee level. First, one person from each business completed the online Healthy Workplace Assessment™ through the Health Links website—www.healthlinkscertified.org—with consensus from team members including the health and safety team [5]. The respondents represent individuals in senior leadership, management, human resources, health and safety, and administration. The assessment contains 35 questions and takes approximately 30–60 min to complete. The assessment was developed based on measures from the Centers for Disease Control and Prevention Worksite Health ScoreCard, the National Institute for Occupational Safety and Health Total Worker Health model, the World Health Organization Healthy Workplace Framework, and a growing body of scientific research [5]. A full discussion of the Healthy Workplace Assessment can be found in Tenney et al. [5]. Next, employees at each business were given the online employee health and safety culture survey. Our research coordinator worked with the main contact at each business to email the survey link to all employees. First, a unique survey link for the business was generated. Then, the study coordinator sent our business contact an employee recruitment email with a copy of the survey link. Then, the business contact forwarded the email to all of their employees. The survey was open for two weeks, and a reminder email was sent one week before closing. If employees completed the survey, they were offered the opportunity to enter a raffle to win one of 15 $100 gift cards. No identifying information was collected in the survey, and the employer was blinded to the individual-level participation and responses.

#### 2.2.1. TWH Strategies

The Health Links Healthy Workplace Assessment scores organizations across six benchmarks to evaluate TWH strategies: (1) organizational support (30 maximum points), (2) workplace assessment (12 maximum points), (3) health policies and programs (16 maximum points), (4) safety policies and programs (16 maximum points), (5) engagement (16 maximum points), and (6) evaluation (10 maximum points) [1]. We calculated a total score (100 maximum points), which represented a sum of all benchmark scores. Employer demographics were also collected from the assessment. The questionnaire collects information on how businesses are implementing health and safety through education, policies, leadership/management commitment, and in certain specific areas such as tobacco control, chronic disease prevention, mental health, ergonomics, and emergency preparedness. Questions asked in each benchmark measure policies and practices that are being currently implemented at the organization. An example question for organizational support is: “In the last 12 months, what resources have you dedicated to workplace health and safety?”

#### 2.2.2. Safety and Health Climates

We used Lee et al.’s [32] 6-item organizational commitment to safety scale to measure safety climate. These questions were prefaced with the following statement: “Please indicate how much you agree or disagree with the following statements related to safety. Safety means preventing you from being injured or made ill on the job”. An example question asked in this section is: “My organization reacts quickly to solve the problem when told about safety concerns”.

We used Zweber et al.’s [18] 4-item organizational commitment to health and well-being scale to measure health climate. These questions were prefaced with the following statement: “Please indicate how much you agree or disagree with the following statements related to your health and well-being. Health and well-being refer to your physical, mental, and emotional health, and their impact on your ability to work”. An example question from this section is: “My organization is committed to employee health and well-being”. Both scales have been tested with prior samples and found to be valid and reliable [18,32].

#### 2.2.3. Leadership Commitment

The leadership commitment questions were developed by the authors and represent overall perceptions of leadership commitment to TWH. Questions asked about leaders’ communication, role modeling, employee recognition, resource allocation, and accountability. These questions mirrored leadership questions measured in the Health Links Healthy Workplace assessment [1]. Leadership commitment to safety questions (5 items) were prefaced by the following statement: “Please indicate how much you agree or disagree with the following statements about your organization’s leadership commitment to safety (preventing you from being injured or made ill on the job): The leaders in your organization are top management and supervisors”. Leadership commitment to worksite wellness questions (5 items) were prefaced by the following statement: “Please indicate how much you agree or disagree with the following statements about your organization’s leadership commitment to worksite wellness (policies and programs that help promote your physical and mental health): The leaders in your organization are top management and supervisors”. For these items, we chose to use the word “safety” to represent health protecting leadership and “worksite wellness” to represent health-promoting leadership because they are commonly understood terms among employees [4].

### 2.3. Analysis

We first evaluated the construct validity and reliability of our measures. We calculated the correlations between all variables and the internal consistency reliability (Cronbach α) of the climate and leadership measures. Then, we ran a confirmatory factor analysis to confirm our hypothesized four-factor structure of safety climate, health climate, leadership commitment to safety, and leadership commitment to worksite wellness using Stata version 14.2 (College Station, TX, USA). We evaluated two competing, nested models: (1) a single-factor model and (2) a two-factor model representing a climate factor and a leadership factor. We used the following goodness-of-fit indices to identify the model that best fit the data: root-mean-squared error of approximation (RMSEA) (<0.08 indicates adequate fit), comparative fit index (CFI) (>0.90 indicates adequate fit), standardized root mean square residual (SRMR) (<0.08 indicates adequate fit), chi-square test, and chi-square difference test between the hypothesized four-factor model and the two competing, nested models [33,34].

Multivariable analyses were performed using linear mixed model regression. We included a random term for business in each of the models to account for correlation between individuals within the same company, and all models were adjusted for tenure, job level, and number of employees the business had. To address hypothesis 1, we first examined the association between the total benchmark score with each of the two climate measures independently. Then, we regressed each of the two climate measures on the total benchmark score (model 1). To address the moderating effect of leadership commitment proposed in hypothesis 2, we re-ran the models with the addition of leadership commitment to safety for safety climate and leadership commitment to worksite wellness for the health climate along with its interaction with the benchmark variable (model 2). All regression analyses were carried out using SAS version 9.4 (Cary, NC, USA).

## 3. Results

### 3.1. Sample

We analyzed the results of 53 businesses representing 4224 employees. Fifty-three percent (53%) of the businesses in our study were in the service industry. The remaining businesses operated in a variety of other industries, including healthcare (11%), public administration (13%), retail and wholesale trade (8%), construction (6%), manufacturing (6%), and transportation (3%). About one-quarter (26%) of the businesses operated in a rural region. Businesses had an average of 80 employees (SD = 98; median 40, range = 16–113).

We collected surveys from a total of 1271 employees. The average response rate per business was 27%. The average age of the survey respondents was 41 (SD = 13), and approximately two-thirds were female (see Table 1). The majority indicated they were White (80%), and 10% reported they were Hispanic. Two-thirds had at least a 4-year college degree, half had a household income greater than $70,000, and nearly 40% worked in a supervisory role. The type of work that the employees engaged in varied, including hourly paid (47%) and shift work (14%).

### 3.2. Confirmatory Factor Analysis

The confirmatory factor analysis of our climate and leadership questions indicated that our hypothesized 4-factor model fit the data best (see Table 2). The CFI, SRMR, and RMSEA indices all indicated good fit [34]. Also, the chi-square difference test between the hypothesized four-factor model and the two alternative models indicated that the four-factor model fit the data best. The standardized loadings of each indicator to its hypothesized construct all exceeded a 0.75 value. These findings provide evidence that these four factors are empirically distinct, supporting our conceptual distinctions among the measures.

### 3.3. Bi-Variable Analyses

Table 3 presents the means, standard deviations, and reliabilities (Cronbach’s α) of the study variables and their correlations. There was significant variability in the average scores for each of the variables of interest. For example, the average Health Links total benchmark score was 48 (SD = 19) out of a possible 100. All study variables were positively correlated with each other. The total benchmark score was positively associated with safety climate (*r* = 0.16, *p* < 0.01) and health climate (*r* = 0.16, *p* < 0.01). Both safety climate and leadership commitment to safety (*r* = 0.77, *p* < 0.01) and health climate and leadership commitment to worksite wellness (*r* = 0.76, *p* < 0.01) exhibited a moderately strong correlation. There were somewhat weaker albeit still statistically significant correlations between the total benchmark score and all other variables.

### 3.4. Regression Analyses–Safety Climate

The total benchmark score was significantly related to safety climate. For every 10-point increase in the total benchmark score, there was a 0.10 increase in safety climate perceptions (on a 1 to 5 scale).

The results presented in Table 4 under model 2 do not support the hypothesis that the relationship between TWH strategy and safety climate is moderated by leadership commitment to safety. After accounting for leadership commitment to safety, the total benchmark score was not significantly related to safety climate. Instead, we observed that for every one-unit increase in leadership commitment to safety there was a 0.66 increase in employee perceptions of safety climate.

### 3.5. Regression Analyses–Health Climate

The total benchmark score was significantly related to health climate such that for every 10-point increase in the total benchmark score there was a 0.10 increase in health climate perceptions (on a 1 to 5 scale).

**Figure 3:**
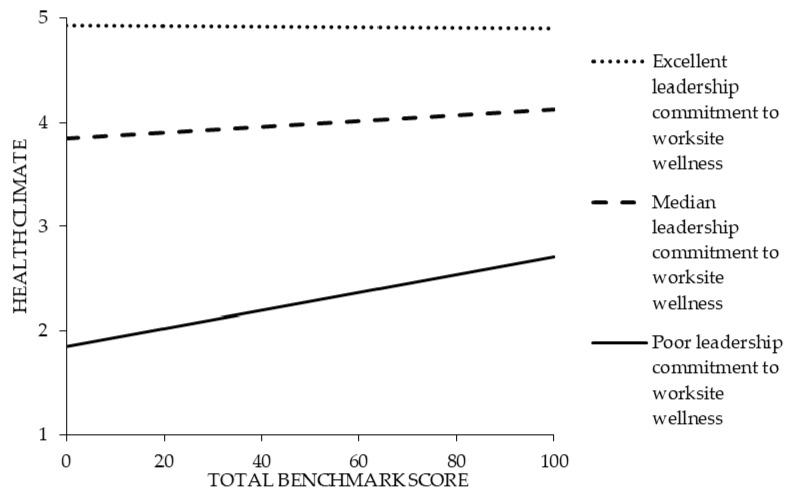
The interactive relationship between total benchmark score and leadership commitment to worksite wellness on health climate.

The results presented in Table 4 under model 2 partially support the hypothesis that the relationship between TWH strategies and health climate is moderated by leadership commitment to worksite wellness. In other words, the relationship between TWH strategies and health climate is dependent on the level of leadership commitment to worksite wellness. This relationship primarily manifested itself under working conditions of low leadership. Specifically, under working conditions where there was a low total benchmark score and poor leadership commitment to worksite wellness, employees reported the worst health climate (see Figure 3). However, under conditions where there was at least a high total benchmark score, employees reported better health climate despite working under conditions of poor leadership commitment to worksite wellness. Under working conditions where leadership commitment to worksite wellness were at median and excellent levels, the effect of the total benchmark score on health climate was minimal.

## 4. Discussion

The purpose of this study was to investigate the relationship of small business TWH strategies and perceptions of leadership on safety and health climates. Our study of 53 small businesses confirmed the importance of the relationship between leadership commitment and workplace safety. In contrast, the relationship between TWH strategies and health climate depended on the level of leadership commitment to worksite wellness. These findings have relevance for how we evaluate organizational climate, since our study demonstrates the value of assessing multiple climates in an organization [9]. Additionally, we were able to demonstrate both similarities and differences in the factors associated with safety climate and health climate. Similar to others [17], we show that safety climate and health climate are related but conceptually distinct constructs, and we further this research by showing that they have a common correlate: leadership commitment to business practices that support either safety or worksite wellness.

Our safety climate results are consistent with previous research demonstrating the association between leadership commitment to safety and safety climate [13,14,25], and we extend these observations to the understanding of small business climate. While prior safety climate research suggests that business policies and programs are related to safety climate [23], we show that in the context of TWH, they were no longer related to employees’ perceptions of safety climate once leadership commitment to safety was accounted for. This suggests that if the goal is to improve safety climate, it is important for businesses to implement strategies to enhance leadership commitment. Taken in context, these findings do not negate the potential importance of TWH strategies in protecting worker health to prevent injuries, illnesses, and fatalities. Several researchers describe conceptual models of how a combination of effective safety and health business strategies and a strong safety climate are needed to enhance safety performance [8,35]. Given our findings, we hypothesize that TWH strategies and safety climate may work independently to influence safety performance. Thus, a next step in this research is to prospectively evaluate these relationships.

To our knowledge, ours is the first study to characterize the associations between TWH strategies and leadership commitment and health climate. Prior research focuses on the relationship between TWH strategies and leadership and *health outcomes*. Previous research links some TWH strategies to health outcomes [36]. While there is meta-analytic evidence that general leadership style is linked to several employee health outcomes such as well-being and the use of sick leave [37], researchers have only recently begun to study health-promoting leadership [38,39,40]. Finally, recent research notes the positive relationship between leadership commitment and worksite wellness program participation and health behavior [41].

We contribute to this literature by showing that both TWH strategies and leadership commitment are associated with health climate. In fact, we found that the level of leadership commitment to worksite wellness moderates the relationship between an organization’s TWH strategies and its health climate. Specifically, poor leadership commitment seemed to have less of an effect on health climate in businesses that were implementing more TWH policies and practices. However, in cases where leadership commitment was strong, the level of TWH strategies were less associated with health climate. These findings are important because it might suggest that in organizations where leadership commitment to health is lacking, the value of TWH strategies is still important. These strategies include providing health insurance and paid sick leave to employees; conducting workplace assessments to identify employee needs and interests; addressing stress, tobacco cessation, mental health, and disease prevention through health-promotion programs; implementing safety policies; and effective communication. These findings warrant future prospective research on the effect of leadership on health climate in small businesses that maintain TWH policies and practices.

### 4.1. Future Research

There is a need for further research on the mechanisms of these relationships. First, research is needed to understand how TWH strategies and leadership work in small business. More specifically, we need to better understand how contextual factors such as size, industry, geographical location, and workforce makeup impact TWH strategies and leadership and ultimately how these differences influence safety and health climates. Furthermore, future research should investigate how safety and health climates are related to each other and how they may or may not work together to influence safety and health outcomes [22]. Finally, there is a need to understand the prospective relationships among TWH strategies, leadership commitment, and safety and health climates, as well as other outcomes such as employee health risk factors and behaviors.

This research also points to a need to develop and assess TWH leadership interventions to improve safety and health climates. Our qualitative research with small business leaders demonstrates that they primarily communicate about TWH through the lens of their business, such as talking about how the TWH strategies they support positively contribute to business outcomes [42]. However, they rarely discuss TWH from the perspective of their employee’s health and safety needs or their own personal health and safety behaviors in the workplace. In the leadership literature, researchers argue that leaders must not only provide TWH resources, but they must also be in tune with needs of employees and supportive of employees engaging in activities that protect and promote their health [25,39]. Furthermore, a key aspect of leadership in a TWH context is ensuring that leaders are role modeling safe work practices and also aware of their own attitudes toward health [40]. We are currently evaluating whether a TWH leadership training program results in a change in safety climate and health climate perceptions in small business [11].

### 4.2. Strengths and Limitations

Our diverse study population included 53 small businesses from multiple industries as well as employees representing different backgrounds. It is also the largest study of its kind to look at the relationship between organizational-level TWH practices and employee perceptions of health and safety. However, our study is limited by the fact that businesses elected to participate in the SSWell study and thus may be more motivated to engage in TWH activities than businesses that chose not to participate. However, given the wide variation in climate perceptions and TWH strategies amongst our study sample (see Table 1 and Table 3), it is unlikely that there is a strong selection bias. Our study design is limited by the cross-sectional nature of the data, and thus assumptions of causality cannot be made. Another limitation is that all data were self-reported. Measures of leadership and climate were measuring using the same methods, and thus the common method bias may be present. However, the effect of the common method bias may be overstated, as not all self-reported constructs are correlated and biases that are related to common methods (e.g., social desirability) do not always inflate the correlation between constructs [43]. The Health Links Healthy Workplace Assessment was completed by a single respondent from the business, which presents a potential for information bias. However, we have previously determined that the job role of the person who completes the assessment (e.g., executive, human resources, or health and safety professional) does not affect responses to questions in the assessment [5]. Additionally, the leadership and climate variables came from a self-reported survey voluntarily taken by employees and subject to selection and recall biases. However, it should be noted that our confirmatory factor analyses supported the idea that our measured variables represented leadership and climate constructs well.

## 5. Conclusions

Our study advances the science of TWH and organizational climate research in several ways. Our findings show that TWH strategies are positively associated with safety and health climates in small businesses. However, TWH strategies are no longer related to safety and health climates after accounting for the effect of leadership commitment. Relatedly, our results demonstrate that leadership is a common correlate to both safety climate and health climate. Future research should investigate integrated TWH leadership development strategies as a means of simultaneously improving safety and health climates.

## Figures and Tables

**Table 1:** Demographic characteristics of study sample.

Demographic Characteristic	N	%	Mean	SD
Age			41.3	13.1
Gender				
Male	419	33.1		
Female	842	66.6		
Other	4	0.3		
Race				
White	1184	80.23		
Black or African American	34	2.3		
Asian	30	2.0		
Native American or Alaskan Native	21	1.4		
Native Hawaiian or Other Pacific Islander	7	0.5		
Ethnicity				
Hispanic or Latino or Spanish Origin	123	9.78		
Not Hispanic or Latino or Spanish Origin	1135	90.2		
Education				
Did not complete high school	8	0.7		
High school diploma or GED	110	9.9		
Some college or 2-year degree	288	25.8		
4-year college degree	462	41.4		
Graduate or professional degree	247	22.2		
Job Level				
Supervisor	500	39.4		
Non-supervisor	768	60.6		
Job Tenure (years)			5.3	6.7
Household income				
<$50,000	396	36.0		
$50,001–$100,000	351	31.9		
>$100,000	353	32.1		
Type of Work				
Full-time	1090	86.4		
Part-time	171	13.6		
Work hours per week			39.4	12.4
Salaried employment	661	52.4		
Hourly employment	600	47.6		
Contractor or consultant	48	3.8		
Shift work	181	14.4		

**Table 2:** Confirmatory factor analysis—goodness of fit indices.

Model	χ^2^	Df	χ^2^_diff_	Df _diff_	CFI	SRMR	RMSEA (90% CI)
*Hypothesized 4 Factors*	*1057*	*164*	*-*	*-*	*0.95*	*0.03*	*0.07 (0.07–0.08)*
Alternative 1 Factor	5140	170	4083	6 *	0.73	0.09	0.17 (0.16–0.17)
Alternative 2 Factors	4105	169	3048	5 *	0.79	0.09	0.15 (0.15–0.15)

Df: Degrees of freedom, CFI: Comparative fit index, SRMR: Standardized root mean square residual, RMSEA: Root-mean-square error of approximation. Alternative 1 factor model: All items are represented by one construct. Alternative 2-factor model: All safety and health climate items are represented by one climate construct, and all leadership commitment to safety and worksite wellness items are represented by one leadership construct. * *p* < 0.01.

**Table 3:** Means, standard deviations, reliabilities, and correlations of TWH strategy, safety and health climates, and health and safety leadership commitments.

Variable	Mean	SD	Safety Climate	Health Climate	Leadership Commitment to Safety	Leadership Commitment to Worksite Wellness	Total Benchmark Score
**Climate**							
Safety climate	3.83	0.79	(0.92)	0.63 *	0.77 *	0.56 *	0.16 *
Health climate	3.88	0.82		(0.86)	0.61 *	0.76 *	0.16 *
**Leadership commitment**							
Leadership commitment to safety	3.68	0.84			(0.90)	0.69 *	0.10 *
Leadership commitment to worksite wellness	3.49	0.91				(0.94)	0.11 *
**TWH strategy**							
Total benchmark score	48.07	18.96					-

Note. Reliabilities (Cronbach alpha) are along the diagonal. Correlations between measures are above the diagonal. * *p* < 0.01.

**Table 4:** Linear mixed model comparing the relationship between total benchmark score (IV) and safety climate/health climates (DVs) as well as the moderating effect of leadership commitment to safety/worksite wellness.

Variable	Model 1		Model 2	
	Estimate	95% CI	Estimate	95% CI
	**Safety climate (DV)**			
Total benchmark score	0.01 *	(0.00, 0.01)	0.00	(−0.01, 0.01)
Leadership commitment to safety			0.66 ***	(0.56, 0.76)
Total benchmark score * Leadership commitment to safety			0.00	(−0.00, 0.00)
	**Health climate (DV)**			
Total benchmark score	0.01 **	(0.00, 0.02)	0.01 **	(0.00, 0.02)
Leadership commitment to worksite wellness			0.77 ***	(0.67, 0.86)
Total benchmark score * Leadership commitment to worksite wellness			−0.002 *	(−0.004, −0.000)

*Note.* All models controlled for tenure, job level, and business size. *** *p* < 0.001. ** *p* < 0.01. * *p* < 0.05. IV = independent variable, DV = dependent variable.
